# Value of Pretreatment ^18^F-fluorodeoxyglucose Positron Emission Tomography in Patients With Localized Pancreatic Cancer Treated With Neoadjuvant Therapy

**DOI:** 10.3389/fonc.2020.00500

**Published:** 2020-04-17

**Authors:** Chad A. Barnes, Mohammed Aldakkak, Callisia N. Clarke, Kathleen K. Christians, Daniel Bucklan, Michael Holt, Parag Tolat, Paul S. Ritch, Ben George, William A. Hall, Beth A. Erickson, Douglas B. Evans, Susan Tsai

**Affiliations:** ^1^LaBahn Pancreatic Cancer Program, Department of Surgery, The Medical College of Wisconsin, Milwaukee, WI, United States; ^2^Department of Radiology, The Medical College of Wisconsin, Milwaukee, WI, United States; ^3^Department of Medicine, Division of Hematology and Oncology, The Medical College of Wisconsin, Milwaukee, WI, United States; ^4^Department of Radiation Oncology, The Medical College of Wisconsin, Milwaukee, WI, United States

**Keywords:** pancreatic cancer, neoadjuvant therapy, carbohydrate antigen 19-9, ^18^F-fluorodeoxyglucose positron emission tomography, SUV

## Abstract

**Background:** ^18^F-fluorodeoxyglucose positron emission tomography/computed tomography (PET/CT) imaging is not routine in patients with localized pancreatic cancer (PC). We evaluated the prognostic value of PET/CT in patients who received neoadjuvant therapy.

**Methods:** Patients with localized PC underwent pretreatment PET/CT with or without posttreatment (preop) PET/CT. Maximum standardized uptake values (SUV) were classified as high or low based on a cut point of 7.5 at diagnosis (SUV_dx_) and 3.5 after neoadjuvant therapy (preoperative; SUV_preop_). Preop carbohydrate antigen 19-9 (CA19-9) was classified as normal ( ≤ 35 U/mL) or elevated.

**Results:** Pretreatment PET/CT imaging was performed on 201 consecutive patients; SUV_dx_ was high in 98 (49%) and low in 103 (51%). Preop PET/CT was available in 104 (52%) of the 201 patients; SUV_preop_ was high in 60 (58%) and low in 44 (42%). Following neoadjuvant therapy, preop CA19-9 was normal in 90 (45%) patients and elevated in 111 (55%). Median overall survival (OS) of all patients was 27 months; 33 months for the 103 patients with a low SUV_dx_ and 22 months for the 98 patients with a high SUV_dx_ (*p* = 0.03). Median OS for patients with low SUV_dx_/normal preop CA19-9, high SUV_dx_/normal preop CA19-9, low SUV_dx_/elevated preop CA19-9, and high SUV_dx_/elevated preop CA19-9 were 66, 34, 23, and 17 months, respectively (*p* < 0.0001). OS was 44 months for the 148 (74%) patients who completed all intended neoadjuvant therapy and surgery and 13 months for the 53 (26%) who did not undergo surgery (*p* < 0.001).

**Conclusion:** Pretreatment PET/CT avidity and preop CA19-9 are clinically significant prognostic markers in patients with PC.

## Introduction

^18^F-fluorodeoxyglucose positron emission tomography/computed tomography (PET/CT) is a diagnostic test that is frequently used to detect distant metastases as an adjunct to cross-sectional imaging in patients with solid tumors (1, 2). Beyond improvements in staging, PET/CT may provide important insights into tumor biology. The maximum standardized uptake values (SUV) obtained with PET/CT reflect the tumor's glucose metabolism; higher tumor SUV has been correlated with aggressive tumor biology and high grade histology (3, 4). Furthermore, decreases in SUV with treatment have been associated with pathologic response and improved overall survival (OS) in esophageal cancer (5, 6). In pancreatic cancer (PC), PET/CT is most commonly used to detect distant metastatic disease. The prognostic value of PET/CT in PC has not been well described particularly in patients with localized PC who receive neoadjuvant therapy (7).

In the past decade, there has been a growing acceptance of neoadjuvant therapy for localized PC in the United States. This change in treatment sequencing arose from two reproducible clinical observations: first, in the absence of systemic therapy, median disease free survival is only 7 months after surgery; and second, there has been a failure to deliver postoperative (adjuvant) therapy in ~50% of eligible patients (8–10). Both of these challenges can be addressed through neoadjuvant treatment sequencing, as systemic therapy for the treatment of radiographically occult metastases can be reliably delivered prior to surgery. Importantly, patients who experience disease progression during or after neoadjuvant therapy can be identified prior to operation—an operation which would provide no therapeutic benefit in the setting of extrapancreatic metastatic disease (11). For those patients who receive neoadjuvant therapy and surgery, median OS (44 months) has been unprecedented when compared to a surgery first approach (12–15). As the experience with neoadjuvant therapy continues to mature, additional prognostic markers are needed to optimize neoadjuvant treatment sequencing. We hypothesize that PET/CT avidity, as measured by the SUV of the primary tumor, may be a surrogate marker of tumor biology. In the current study, we evaluated the prognostic value of pretreatment SUV (SUV_dx_) and preoperative SUV (SUV_preop_) on OS among patients with localized PC treated with neoadjuvant therapy.

## Methods

### Study Subjects

This study was approved by the Institutional Review Board at the Medical College of Wisconsin. Using a prospectively maintained database, consecutive patients were identified between 2009 and 2017 who had histologically confirmed localized PC and underwent PET/CT imaging as part of their routine clinical care at the time of diagnosis. Patients met objective radiographic criteria for either resectable or borderline resectable (BLR) PC (16) Patients were also classified as BLR if there were radiographic findings indeterminate for metastases and/or the serum CA19-9 level at diagnosis was >2,000 U/mL. Serum CA19-9 levels were considered evaluable if measured when the serum bilirubin was ≤ 2 mg/dL and were categorized as normal ( ≤ 35 U/ml) or elevated (>35 U/mL).

#### Neoadjuvant Therapy and Surgery

All patients received neoadjuvant therapy consisting of either chemotherapy alone, chemoradiation, or both. The majority of patients with resectable PC received chemotherapy alone or gemcitabine-based chemoradiation as neoadjuvant therapy. The majority of patients with BLR PC were treated with a minimum of 2 months of induction chemotherapy followed by either gemcitabine- or capecitabine-based chemoradiation. Approximately 4 weeks following the completion of neoadjuvant therapy, preoperative (preop) restaging was performed and consisted of a history and physical examination, CT scan, PET/CT, and laboratory studies. In some patients, preop PET/CT was often limited by insurance approval if a pretreatment PET had been performed. Patients who demonstrated disease progression or had an inadequate performance status at the time of restaging were not offered surgery. Following surgical resection, follow-up occurred at 3–4 month intervals with physical examination, laboratory studies, and CT imaging. Disease recurrence was assessed radiographically and in select cases, confirmed with tissue biopsy.

### PET/CT Technique and Review

**Figure 1:**
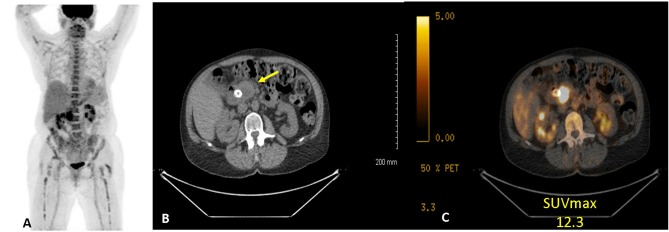
Example of ^18^FDG PET/CT of a women with PC. **(A)** anterior maximum intensity projection, **(B)** axial CT, and **(C)** axial fused CT. Study demonstrates a hypermetabolic (SUV_dx_ 12.3) primary pancreatic cancer.

PET/CT imaging was performed using a GE Discovery 710 (GE Healthcare, Waukesha, WI) scanner. In preparation for image acquisition, patients were instructed to fast for 4–6 h and to refrain from any strenuous activity for 2 days prior to the examination. Blood glucose levels were measured 1–2 h prior to imaging. If a patient's blood glucose was greater than 200 mg/dL, PET/CT imaging was rescheduled after the optimization of glycemic control. On the day of image acquisition, patients were injected with the FDG radiotracer ~60 min prior to the scan. Intravenous administration of FDG was weight based with a standard dose of 370 megabecquerel (mBq) for patients weighing <55 kilograms (kg), 444 mBq for patients weighing 55–91 kg, and 518 mBq for patients weighing >91 kg. After an incubation period of ~60 min, patients underwent concurrent FDG-PET imaging with a low dose non-contrast CT. Registered noncontrast enhanced axial CT images were obtained through these same levels to use for attenuation correction and were reviewed to localize FDG uptake (Figure 1).

All PET/CT studies were reviewed by a comprehensive team of radiologists, including at least one abdominal imaging radiologist, one fellowship trained nuclear radiologist, and one board certified nuclear medicine physician. Images were analyzed on a diagnostic nuclear radiology reviewer (AW Server; GE Healthcare, Waukesha, WI). FDG uptake was used to localize the primary tumor, regional lymph nodes, and any sites of extrapancreatic metastases. The peak SUV was determined by placing a region of interest around the lesion that was being evaluated. The maximal SUV was defined as the measured activity normalized for body weight/surface area and determined using the following equation: region of interest activity (mBq/mL) x patient body weight (kg)/injected FDG dose (17). SUV_dx_ was categorized as low ( ≤ 7.5) or high (>7.5) based on the population median SUV on pretreatment PET/CT. The SUV_preop_, following neoadjuvant therapy, was categorized as normalized ( ≤ 3.5) or elevated (>3.5) based on prior data which have associated values <3.5 with non-cancerous tissue (18).

### Statistical Analysis

Categorical variables were compared using the Fischer's Exact or Chi-squared test. All continuous variables were analyzed using the Mann-Whitney U test. Overall survival (OS) and follow-up was calculated from the time of initial diagnosis to the date of death or last follow-up. Deaths from any cause were included in the survival analysis. OS was estimated using the method of Kaplan and Meier. We tested proportional hazard assumptions for all variables associated with survival. Clinical factors with a univariable *p* < 0.20 were included in the multivariable model. All statistical analyses were performed using Stata 14.2 (StataCorp, College Station, Texas).

## Results

### Patient Characteristics

We identified 211 consecutive patients with localized PC who underwent pretreatment PET/CT imaging prior to the initiation of neoadjuvant therapy. Of the 211 patients, 10 did not have a SUV reported and were excluded. Of the remaining 201 patients, 88 (44%) had resectable and 113 (56%) had BLR PC. The median age was 66 years [interquartile range (IQR): 13], and 103 (51%) of the patients were male. Patient demographic data is further summarized in Table 1. Pretreatment CA19-9 was evaluable in 163 (81%) of the 201 patients; 42 (26%) had a pretreatment CA19-9 ≤ 35 U/mL and 121 (74%) patients had an elevated pretreatment CA19-9 (median 226 U/mL; IQR: 504).

**Table 1:** Clinicopathologic characteristics of all patients (*n* = 201).

**Variable(s)**	**Total** ***n* = 201**	**Pretreatment** **SUV ≤ 7.5** ***n* = 103**	**Pretreatment** **SUV > 7.5** ***n* = 98**	***p*-value**
Male Gender, *n* (%)	103 (51)	52 (50)	51 (52)	0.83
Age, median (IQR)	66 (13)	65 (13)	66 (14)	0.68
Charlson Comorbidity Index, median (IQR)	5 (2)	5 (2)	5 (2)	0.10
Clinical Stage, *n* (%)				0.16
Resectable	88 (44)	50 (49)	38 (39)	
BLR	113 (56)	53 (51)	60 (61)	
Pretreatment CA19-9, median (IQR)	226 (504)	161 (481)	322 (652)	0.08
Pretreatment PET SUV, median (IQR)	7.5 (3.9)	5.6 (2)	9.5 (3.7)	<0.0001
Neoadjuvant Therapy, *n* (%)				0.45
Chemotherapy alone	41 (20)	22 (21)	19 (19)	
Chemoradiation	56 (28)	32 (31)	24 (25)	
Both	104 (52)	49 (48)	55 (56)	
Preop CA19-9, median (IQR)	43 (98)	41 (67)	44 (141)	0.73
% change in CA19-9^¥^	−52	−52	−54	1.00
Normalization of Preop CA19-9				0.97
Normal	90 (45)	46 (45)	44 (45)	
Elevated	111 (55)	57 (55)	54 (55)	
Preop PET available, *n* (%)	104 (52)	56 (54)	48 (49)	0.48
Preop PET SUV, median (IQR)^Δ^	4.2 (3.7)	3.4 (4.9)	4.5 (3.4)	0.08
% change in SUV^Δ^	−49	−44	−53	0.24
Surgery, *n* (%)				0.49
No	53 (26)	25 (24)	28 (29)	
Yes	148 (74)	78 (76)	70 (71)	

¥ *Among the 163 patients with evaluable CA19-9 at diagnosis, ^Δ^ Among the 104 patients with restaging preoperative PET scans*.

### Pretreatment PET/CT SUV

Of the 201 patients with pretreatment PET/CT imaging, 175 (87%) underwent imaging at our institution. The median serum glucose level prior to intravenous injection of FDG was 113 (IQR: 45). The median SUV_dx_ of the primary tumor was 7.5 (IQR:3.9); 103 (51%) patients had a low SUV ( ≤ 7.5) and 98 (49%) had a high SUV (>7.5). Eight (4%) patients had a SUV_dx_ ≤ 3.5. Overall, there was no association between clinical stage and SUV_dx_ level. However, of the 113 patients with BLR disease, SUV_dx_ levels in the primary tumor were higher in the 21 (19%) patients with radiographic findings indeterminate for metastases as compared to the 92 (81%) patients without indeterminate lesions, (9.0 vs. 8.5, respectively, *p* = 0.05).

### Neoadjuvant Therapy and Preoperative Restaging

All 201 patients received neoadjuvant therapy; 41 (20%) patients had chemotherapy alone, 56 (28%) had chemoradiation alone, and 104 (52%) had chemotherapy followed by chemoradiation. SUV_dx_ levels were not associated with the type of neoadjuvant therapy received (*p* = 0.45). Following the completion of neoadjuvant therapy, preop CA19-9 levels were measured in all patients; 111 (55%) had an elevated preop CA19-9 and 90 (45%) had a normal preop CA19-9. Of the 121 patients with an elevated pretreatment CA19-9, 42 (35%) declined to a normal range following neoadjuvant therapy. SUV_dx_ was not associated with normalization of preop CA19-9 level [odds ratio (OR): 1.09, 95% confidence interval (CI): 0.51–2.32].

### Change in SUV With Neoadjuvant Therapy

Restaging, preop PET/CT was obtained in 104 (52%) of the 201 patients; 44 (42%) had a normal SUV_preop_ ( ≤ 3.5) and 60 (58%) had an elevated SUV_preop_ (>3.5). The median SUV_preop_ was 4.2 (IQR: 3.7) and the median proportional change in SUV during treatment was−49% (IQR: 51%). Of the 104 patients, 56 (54%) originally had low SUV_dx_ ( ≤ 7.5) and 48 (46%) had high SUV_dx_ (>7.5). Of the 56 patients with low SUV_dx_, 29 (52%) normalized their SUV_preop_ and 27 (48%) remained elevated. Of the 48 patients with high SUV_dx_, 15 (31%) normalized their SUV_preop_ and 33 (69%) remained elevated. Patients with a low SUV_dx_ were more likely to have a normal SUV_preop_ following neoadjuvant therapy than patients with a high SUV_dx_ (*p* = 0.04).

### Preop SUV and Preop CA19-9

Patients with an elevated SUV_preop_ had an increased odds of having an elevated preop CA19-9. Of the 44 patients with a normal SUV_preop_, 26 (59%) had a normal preop CA19-9 and 18 (41%) had an elevated preop CA199. Of the 60 patients with an elevated SUV_preop_, 21 (35%) had a normal preop CA19-9 and 39 (65%) had an elevated preop CA19-9. In an adjusted logistic regression analysis, patients with an elevated preop CA19-9 were more likely to have an elevated SUV_preop_ (OR: 2.69; 95% CI: 1.19–6.11; Table 2) as compared to patients with a normal preop CA19-9.

**Table 2:** Logistic regression analysis for elevated preop SUV (*n* = 104).

**Variable(s)**	**Univariable**			**Multivariable**		
	**OR**	**95% CI**	***p*-value**	**OR**	**95% CI**	***p*-value**
Age						
≤ 65 years		Reference			Reference	
>65 years	0.80	0.36–1.78	0.59	0.72	0.30–75	0.47
Gender						
Female		Reference			Reference	
Male	0.99	0.45–2.18	0.99	1.00	0.44–2.28	0.99
Age-adjusted CCI						
≤ 5		Reference			Reference	
>5	0.97	0.41–2.28	0.94	1.00	0.39–2.60	0.99
Clinical Stage						
Resectable		Reference			Reference	
BLR	1.47	0.67–3.20	0.34	1.56	0.69−3.51	0.29
Preop CA19-9						
Normal ( ≤ 35 U/mL)		Reference			Reference	
Elevated (>35 U/mL)	2.68	1.20–5.98	0.02	2.69	1.19–6.11	0.02

### Surgery and Pathologic Features

All intended neoadjuvant therapy and surgery was completed in 148 (74%) of the 201 patients; 53 (26%) patients did not undergo pancreatectomy. Completion of neoadjuvant therapy and surgery occurred in 83 (80%) of the 104 patients with a preop PET/CT. The most common operation was a pancreaticoduodenectomy (*n* = 116; 78%). Surgery was not performed in 53 patients due to disease progression at the time of preoperative restaging (*n* = 31; 15%), positive diagnostic laparoscopy (11; 5%), poor performance status (9; 4%), or the patient declined surgery (2; 1%).

The pathologic details for the 148 resected patients are summarized in Table 3. The majority of tumors were T3 (*n* = 103, 70%) and were well or moderately differentiated (*n* = 110, 80%). Histologic grade was associated with SUV_dx_ activity; 17 (24%) of the 70 patients with high SUV_dx_ tumors had poorly differentiated tumors as compared to 8 (10%) of the 78 patients with low SUV_dx_ tumors (*p* = 0.03). Patients with a normal SUV_preop_ were more likely to have smaller tumors than patients with an elevated SUV_preop_ (2.0 vs. 2.8, *p* = 0.06).

**Table 3:** Clinicopathologic characteristics of resected patients (*n* = 148).

**Variable(s)**	**Total** ***n* = 148**	**Pretreatment** **SUV ≤ 7.5** ***n* = 78**	**Pretreatment** **SUV > 7.5** ***n* = 70**	***p*-value**
T Stage, *n* (%)				0.32
T0	6 (4)	2 (2)	4 (6)	
T1	12 (8)	9 (12)	3 (4)	
T2	27 (18)	15 (19)	12 (17)	
T3	103 (70)	52 (67)	51 (73)	
N Stage, *n* (%)				0.21
N0	85 (57)	41 (53)	44 (63)	
N1	63 (43)	37 (47)	26 (37)	
Median tumor size, cm (IQR)	2.5 (1.6)	2.6 (1.5)	2.5 (1.7)	0.66
Poorly to undifferentiated tumor, *n* (%)	25 (17)	8 (10)	17 (24)	0.03
Pathologic response, *n* (%)^¥^				0.92
CR or Near CR	27 (18)	14 (18)	13 (19)	
PR or no response	121 (82)	64 (82)	57 (81)	
Perineural invasion, *n* (%)	100 (68)	58 (74)	42 (60)	0.06
Lymphovascular invasion, *n* (%)	39 (26)	21 (27)	18 (26)	0.88
Positive margin, *n* (%)	22 (15)	11 (14)	11 (16)	0.78
Elevated postoperative CA19-9, *n* (%)	37 (25)	19 (24)	18 (26)	0.55
Adjuvant therapy, *n* (%)	99 (68)	62 (82)	37(53)	<0.001

¥ *CR, Complete response, PR, Partial response*.

### Pretreatment SUV and Overall Survival

**Figure 2:**
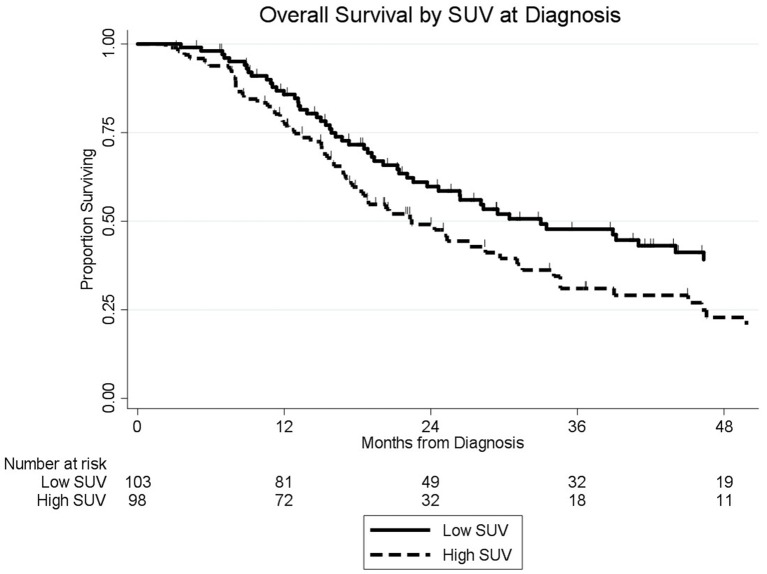
Overall survival by pretreatment SUV level (*n* = 201).

**Figure 3:**
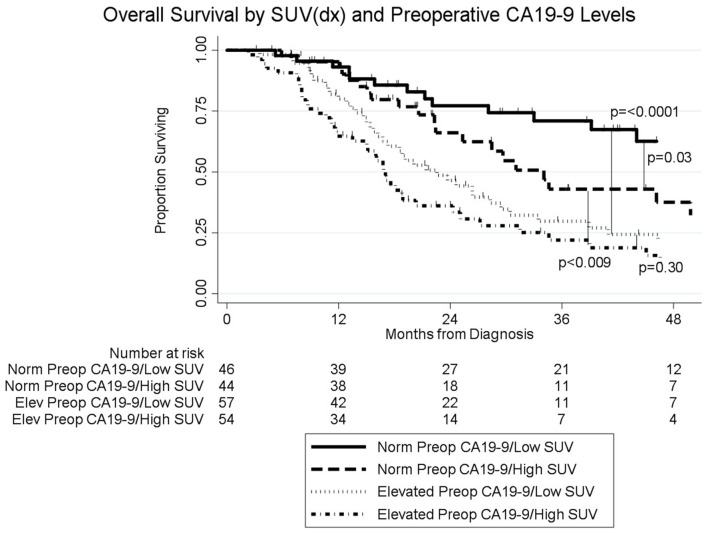
Overall survival by pretreatment SUV and preop CA19-9 levels (*n* = 201).

The median follow-up of all living patients was 55 months.The median OS was 27 months for all 201 patients; 33 months for the 103 patients with a low SUV_dx_ as compared to 22 months for the 98 patients with a high SUV_dx_ (*p* = 0.03; Figure 2). The median OS of the 90 patients with a normal preop CA19-9 was 50 months as compared to 19 months for the 111 patients with an elevated preop CA19-9 (*p* < 0.0001). The median OS for patients with low SUV_dx_/normal preop CA19-9, high SUV_dx_/normal preop CA19-9, low SUV_dx_/elevated preop CA19-9, and high SUV_dx_/elevated preop CA19-9 were 66, 34, 23, and 17 months, respectively (*p* < 0.0001; Figure 3). Low vs. high SUV_dx_ further dichotomized patients who had a normal preop CA19-9 (Figure 3, *p* = 0.03), but not patients who had an elevated preop CA19-9 level. In an adjusted hazards analysis of survival for the 201 patients, clinical stage (HR:1.68; 95%CI:1.16–2.45), SUV_dx_ (HR:1.47; 95%CI:1.02–2.11) and preop CA19-9 (HR:2.57; 95%CI:1.74–3.81) were significant prognostic factors (Table 4). Although normalization of preop CA19-9 was the strongest prognostic factor for OS, the addition of SUV_dx_ further improved the risk estimation. When compared to patients with both low SUV_dx_ and normal preop CA19-9 levels, patients with high SUV_dx_ and elevated preop CA19-9 had a 3.8-fold increased risk of death (95%CI: 2.14–6.87, *p* < 0.001); those with a low SUV_dx_ and an elevated preop CA19-9 had a 2.67-fold increased risk of death (95%CI: 1.49–4.81, *p* = 0.001); and those with a high SUV_dx_ and a normal preop CA19-9 had a 1.54-fold increased risk of death (95%CI: 0.80–2.97, *p* = 0.20). For the 148 patients who completed all neoadjuvant therapy and surgery, the median OS was 44 months (range 5–99 months) compared to 13 months (range 2–39 months) for the 53 patients who were not resected (*p* < 0.0001).

**Table 4:** Cox proportional hazards analysis (*n* = 201).

**Variable(s)**	**Univariable**			**Multivariable**		
	**HR**	**95% CI**	***p*-value**	**HR**	**95% CI**	***p*-value**
Age						
≤ 65 years		Reference			Reference	
>65 years	0.90	0.62–1.30	0.58	–	–	–
Gender						
Female		Reference			Reference	
Male	0.97	0.67–1.39	0.85	–	–	–
Age-adjusted CCI						
≤ 5		Reference			Reference	
>5	1.16	0.79–1.70	0.46	–	–	–
Clinical Stage						
Resectable		Reference			Reference	
Borderline Resectable	1.64	1.12–2.38	0.009	1.68	1.16–2.45	0.006
Neoadjuvant Therapy						
Chemoradiation		Reference			Reference	
Chemotherapy	0.98	0.58–1.66	0.95	–	–	–
Both	1.02	0.67–1.57	0.91	–	–	–
Pretreatment SUV						
Low ( ≤ 7.5)		Reference			Reference	
High (>7.5)	1.49	1.04–2.15	0.03	1.47	1.02–2.11	0.04
Preop CA19-9						
Normal ( ≤ 35 U/mL)		Reference			Reference	
Elevated (>35 U/mL)	2.45	1.66–3.63	<0.001	2.57	1.74–3.81	<0.001

### Preop SUV and Overall Survival

**Figure 4:**
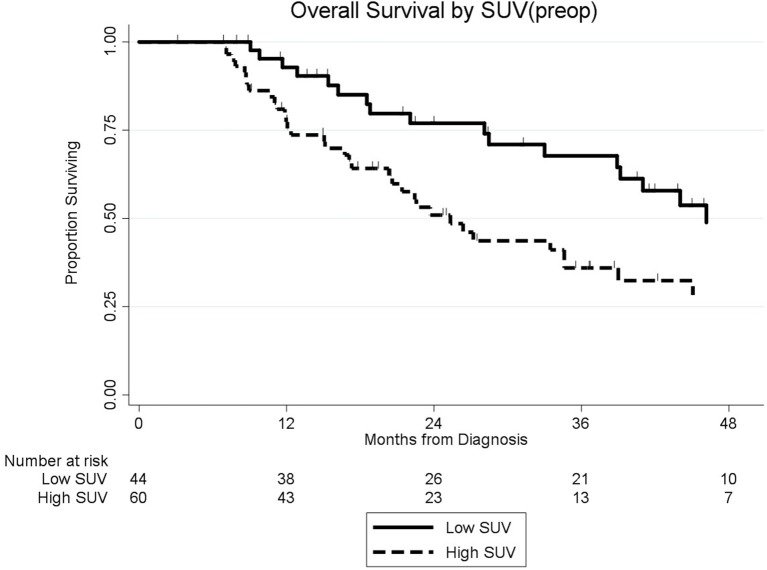
Overall survival by preop SUV level (*n* = 104).

**Figure 5:**
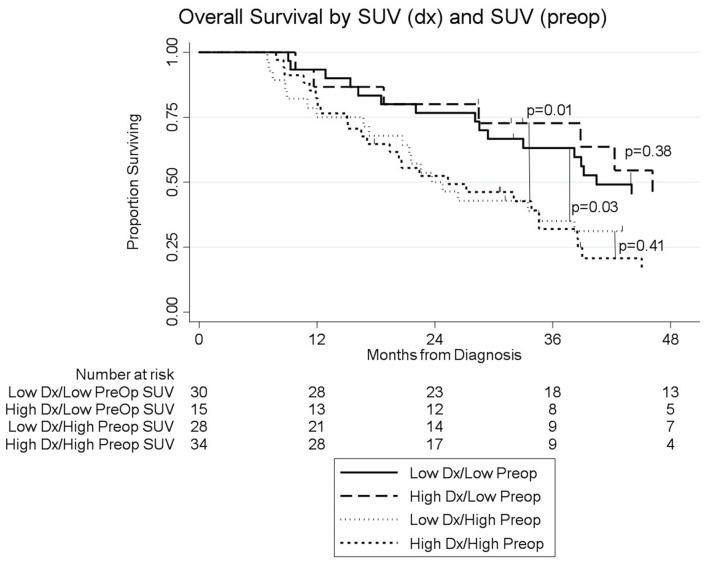
Overall survival by pretreatment and preop SUV status (*n* = 104).

Among the 104 patients with a preop PET/CT, the median OS was 35 months; 46 months for the 44 patients with a normal SUV_preop_ and 25 months for the 60 patients with an elevated SUV_preop_ (*p* = 0.02; Figure 4). When comparing SUV_dx_ and SUV_preop_ levels, OS was driven by SUV_preop_ status rather than SUV_dx_ status. The median OS for the patients with low SUV_dx_/normal SUV_preop_, high SUV_dx_/normal SUV_preop_, low SUV_dx_/elevated SUV_preop_, and high SUV_dx_/elevated SUV_preop_ were 44, 46, 26, and 25 months, respectively (*p* = 0.04; Figure 5). The median OS for patients with normal SUV_preop_/normal preop CA19-9, elevated SUV_preop_/normal preop CA19-9, normal SUV_preop_/elevated preop CA19-9, and elevated SUV_preop_/elevated preop CA19-9 were not reached, 66, 19, and 23 months, respectively (Table 5). However, in an adjusted hazards analysis, while clinical stage (HR:1.94; 95%CI:1.12–3.34) and elevated preop CA19-9 (HR:2.69; 95%CI:1.47–4.92) were negative prognostic factors, elevated SUV_preop_ (HR 1.46; 95%CI: 0.81–2.63) was not.

**Table 5:** Median overall survival by preop SUV and preop CA19-9 (*n* = 104).

	**Normal preop CA19-9** **(<35 U/mL)**	**Elevated preop CA19-9** **(>35 U/mL)**
Low preop SUV ( ≤ 3.5)	OS not reached *n* = 26	19 months *n* = 18
High preop SUV (>3.5)	66 months *n* = 21	23 months *n* = 39

## Discussion

To our knowledge, the current study is the first reported analysis of the prognostic value of PET/CT in patients who received neoadjuvant therapy for operable PC. In a series of 201 consecutive patients, elevated SUV_dx_ was an independent negative prognostic factor for OS and when combined with preop CA19-9 levels, SUV_dx_ status further improved the risk stratification for OS. Following neoadjuvant therapy, preop PET/CT was available in just half (52%) of all patients due to failure of insurance approval for a repeat scan. Importantly, there were significant changes in SUV levels following neoadjuvant therapy (median change −49) and there was a trend toward improved OS for patients who normalized their SUV_preop_. These observations suggest that SUV_dx_ and SUV_preop_ values may be valuable prognostic markers for patients with localized PC who receive neoadjuvant therapy.

Significant improvements have been made in the survival of patients with localized PC. Among patients who are able to complete all intended neoadjuvant therapy and surgery, the median OS is now approaching four years (14, 15). However, early postoperative recurrences still occur and a greater understanding of risk factors associated with early disease recurrence is needed to improve surgical selection and better guide patients in shared-decision making. CA19-9 is a valuable quantitative biomarker in PC which has been correlated with stage of disease and high levels of CA19-9 have been inversely associated with survival (19–23). More recently, dynamic changes in CA19-9 in response to neoadjuvant therapy have also been identified as a powerful prognostic factor (22, 24). We have previously observed a positive association between normalization of CA19-9 levels following neoadjuvant therapy and median OS (19). In the current study, patients with a normal preop CA19-9 experienced a median OS of 50 months as compared to 19 months among patients whose preop CA19-9 remained elevated (*p* < 0.0001).

Importantly, we demonstrated that high SUV_dx_ was an adverse prognostic marker independent of preop CA19-9 level. SUV is a semi-quantitative measure of FDG uptake and the maximal SUV in a primary tumor has been reported to be inversely related to disease progression and survival duration (25–27). FDG uptake by the primary tumor may be a surrogate marker of biologic aggressiveness; high pretreatment SUV levels were associated with poorly differentiated tumors, as the glucose metabolism is accelerated in rapidly proliferating malignant cells (18). In a review by Hu et al. a significant correlation was found between ki-67 index and SUV among 45 patients with PC as compared to those with pancreatic neuroendocrine tumors or cystic neoplasms (*p* < 0.001) (18). In another study which included 102 patients with resected and locally advanced PC, Ahn et al. observed that primary tumor maximal SUV closely correlated with PC histologic grade (25). The mean SUVs for well-, moderately-, and poorly differentiated tumors were 4.93, 6.47, and 7.29, respectively (*p* = 0.009), and there was an inverse relationship between SUV and median OS. Similarly, in pancreatic neuroendocrine carcinomas, FDG-PET uptake has been correlated with higher proliferation (measured by ki-67) and poorly differentiated tumor grade, reflecting an aggressive tumor biology (28, 29). The additional information provided by pretreatment PET SUV levels may provide further insight into the biology of the tumor which is not reflected by quantitative changes in CA19-9.

To our knowledge, this is also the first reported analysis of serial PET imaging in patients with PC before and after neoadjuvant therapy. Our analysis included a subset of 104 patients with both pretreatment and preop PET/CT scans. Neoadjuvant therapy was associated with a significant decline in SUV (~50%) in the majority of patients. Based on our previous experience with CA19-9 monitoring, we chose to dichotomize patients with preop PET/CT based on an SUV value ( ≤ 3.5) associated with normal tissue (18). In the current study, patients with elevated SUV_preop_ levels following neoadjuvant therapy were more likely to also have an elevated preop CA19-9. When comparing patients by the pretreatment and preop SUV levels, patients who had a high SUV_dx_ but normalized the SUV_preop_ ( ≤ 3.5) had a significantly improved OS as compared to patients with a persistently elevated SUV_preop_ (>3.5). However, in a multivariable analysis, the impact of normalization of SUV following neoadjuvant therapy did not meet statistical significance. Nevertheless, this early data provides a signal that the normalization of SUV following neoadjuvant therapy may be a valuable prognostic marker and should be examined in a larger cohort of patients.

There are several limitations of this study. PET/CT is a complicated diagnostic procedure which can yield a variable SUV. Most (>80%), but not all, of the patients included in this study had their PET/CT performed at a single institution using the same diagnostic protocol. This heterogeneity adds to the uncertainty of the what values should be used as appropriate cutpoints for determining meaningful biologic SUV avidity. We chose to utilize the median SUV_dx_ of our cohort to categorize patients as having high vs. low SUV_max_, but chose to utilize a SUV_preop_ of 3.5 based on previously published data regarding normal FDG avidity of the pancreas. The choice of these cutpoints may not be generalizable and should be further refined in future studies. Another limitation of the study was the inability to obtain a preop PET/CT in all patients. Repeat diagnostic imaging become increasingly difficult given the complexity of health insurance in this country. We found no association between normalization of SUV_preop_ and OS, however given the small sample size of patients who had a preop PET/CT, the association between SUV_preop_ and survival should be examined in a larger cohort of patients. Our study utilized data from the clinical PET/CT report and therefore was limited to evaluating the reported maximal SUV. There is a growing field of radiomic analysis that converts imaging data into quantitative elements which can be analyzed to improve diagnostic, prognostic, and predictive accuracy. Radiomic analysis for PET/CT in patients with PC may expand the analysis beyond maximal SUV, to include other metabolic parameters as well as volumetric parameters, such as metabolic tumor volume (MTV) and total lesion glycolysis (TLG). Unfortunately, these parameters are not included in the clinical reports from our PET/CT scans and beyond the scope of the current study. However, these parameters are likely to provide an even greater association of PET/CT results with tumor biology than is reflected in maximal SUV alone. Metabolic parameters, such as maximal SUV, peak SUV, and tumor background ratio have been identified as predictive of venous infiltration in patients in PC (30). Volumetric parameters such as metabolic tumor volume and total lesion glycolysis have been associated with recurrence-free and OS (31). Future studies utilizing a more comprehensive radiomic analysis are needed and may further complement blood-based biomarker analyses.

## Conclusions

Neoadjuvant therapy for PC allows for the immediate delivery of systemic therapy and provides a window of opportunity for clinicians to improve patient selection for surgery. While CA19-9 monitoring mirrors quantitative changes in the burden of disease, SUV_dx_ levels may provide complimentary information in estimating the tumor's biologic behavior. The value of serial SUV monitoring in patients after the completion of neoadjuvant therapy should be an area of further study. The monitoring of dynamic quantitative markers of treatment response such as FDG-avidity and CA19-9 are important surrogate endpoints for the assessment of treatment efficacy as they quantitatively differentiate treatment response from disease stabilization. This will allow physicians to optimize treatment sequencing and improve patient selection for such extensive, and at times, high-risk surgical procedures.

## Data Availability Statement

The datasets generated for this study are available on request to the corresponding author.

## Ethics Statement

The studies involving human participants were reviewed and approved by Medical College of Wisconsin IRB. Informed consent was not required by our IRB. This was a retrospective study of a prospective database that did not require patient consent. We had no individuals under the age of 16.

## Author Contributions

ST and CB: conception, design, analysis, and interpretation. DB, MH, and PT: acquisition of data and analysis. MA, CC, KC, PR, BG, WH, BE, and DE: analysis and interpretation.

## Conflict of Interest

The authors declare that the research was conducted in the absence of any commercial or financial relationships that could be construed as a potential conflict of interest.
